# Effect of joint mobilization techniques for primary total knee arthroplasty: Study protocol for a randomized controlled trial

**DOI:** 10.1097/MD.0000000000008827

**Published:** 2017-12-08

**Authors:** Jiao Xu, Juan Zhang, Xue-Qiang Wang, Xuan-Lin Wang, Ya Wu, Chan-Cheng Chen, Han-Yu Zhang, Zhi-Wan Zhang, Kai-Yi Fan, Qiang Zhu, Zhi-Wei Deng

**Affiliations:** a Sport Medicine and Rehabilitation Center, Shanghai University of Sport; b Department of Rehabilitation Medicine, Shanghai Shangti Orthopedics Hospital, Shanghai, China.

**Keywords:** joint mobilization technique, physical therapy, randomized controlled trial, rehabilitation, total knee arthroplasty

## Abstract

**Background:**

Total knee arthroplasty (TKA) has become the most preferred procedure by patients for the relief of pain caused by knee osteoarthritis. TKA patients aim a speedy recovery after the surgery. Joint mobilization techniques for rehabilitation have been widely used to relieve pain and improve joint mobility. However, relevant randomized controlled trials showing the curative effect of these techniques remain lacking to date. Accordingly, this study aims to investigate whether joint mobilization techniques are valid for primary TKA.

**Methods/Design:**

We will manage a single-blind, prospective, randomized, controlled trial of 120 patients with unilateral TKA. Patients will be randomized into an intervention group, a physical modality therapy group, and a usual care group. The intervention group will undergo joint mobilization manipulation treatment once a day and regular training twice a day for a month. The physical modality therapy group will undergo physical therapy once a day and regular training twice a day for a month. The usual care group will perform regular training twice a day for a month. Primary outcome measures will be based on the visual analog scale, the knee joint Hospital for Special Surgery score, range of motion, surrounded degree, and adverse effect. Secondary indicators will include manual muscle testing, 36-Item Short Form Health Survey, Berg Balance Scale function evaluation, Pittsburgh Sleep Quality Index, proprioception, and muscle morphology. We will direct intention-to-treat analysis if a subject withdraws from the trial.

**Discussion:**

The important features of this trial for joint mobilization techniques in primary TKA are randomization procedures, single-blind, large sample size, and standardized protocol. This study aims to investigate whether joint mobilization techniques are effective for early TKA patients. The result of this study may serve as a guide for TKA patients, medical personnel, and healthcare decision makers.

**Trial registration:**

It has been registered at http://www.chictr.org.cn/showproj.aspx?proj=15262 (Identifier:ChiCTR-IOR-16009192), Registered 11 September 2016. We also could provide the correct URL of the online registry in the WHO Trial Registration. http://apps.who.int/trialsearch/Trial2.aspx?TrialID=ChiCTR-IOR-16009192

## 1 Background

Knee osteoarthritis (OA) is an ordinary degenerative joint disease and a primary cause of dysfunction in the elderly, thereby burdening health resources.^[1,2]^ Total knee arthroplasty (TKA) is recommended to ease the pain and increase the joint function of end-stage knee OA patients. The number of TKA patients in developed countries has increased sharply.^[3–5]^ Statistics show that 93% of knee OA patients experience relieved joint pain, alleviated stiffness, and improved movability after replacement.^[6]^ However, TKA often leaves early postoperative complications, such as pain, restricted joint activities, and muscle atrophy, which cause difficulty in daily life activities and reduce quality of life.^[7,8]^ Physical therapy can hasten the recovery of TKA surgery patients.^[9]^ In the United States, the latest research data show that the mean total TKA-related expenses are US $ 30,831; speeding up the recovery process can lessen TKA-related expenses.^[10]^

Traditional rehabilitation programs^[11]^ primarily aim to improve the knee strength, increase the range of movement, and enhance the gait of TKA patients. Early-stage TKA patients mainly lie in bed to perform straight leg-raising to increase quadriceps muscle strength and active joints.^[12]^ Even TKA patients who completed the traditional rehabilitation training plan still experience reduced walking speed and difficulty in climbing.^[13]^ The persistence of functional limitations signifies the need to find effective rehabilitation strategies for TKA surgery patients.

Joint mobilization techniques for rehabilitation are commonly employed by physical therapists to relieve pain and increase motion in TKA patients.^[14,15]^ Two reasons explain why joint mobilization techniques may be useful for primary TKA. First, early postoperative TKA is associated with pain and restricted range of motion.^[16]^ Joint mobilization may assist in reducing pain and increasing motion by passive oscillatory movements of small or large amplitude and sustained stretching.^[14]^ Second, TKA patients often encounter muscle weakness. Mobilization may accelerate TKA rehabilitation by increasing corticospinal excitability, allowing physiotherapists to optimize muscle recruitment rates and constant movement.^[17]^

A systematic review has shown that early mobilization after a hip or knee arthroplasty can reduce the length of hospital stay to about 1.8 days without any increase in adverse results.^[18]^ Joint mobilization, as a clinical commonly used intervention, can alleviate the chronic pain of knee OA patients by reducing the excitability of reflection.^[19]^ A further study on traction mobilization is important to revise TKA surgery such that the recovery of joint activities is promoted and the incidence of infection is managed.^[20]^ Many studies have reported the role of joint mobilization in the cervical vertebra, lumbar, shoulder, and ankle, but randomized controlled trials (RCTs) showing the effect of joint mobilization on early postoperative TKA rehabilitation remain lacking to date.^[21–23]^

Effective joint mobilization for primary TKA is important to promote the fast and efficient recovery of patients and to reduce economic expenditure. Hence, we project a single-blind RCT to conclude the effect of joint mobilization techniques for primary TKA.

## 2 Methods/design

### 2.1 Research object

We will accomplish an RCT on the effect of joint mobilization techniques for primary TKA to determine the following:

1) Whether mobilization benefits the rehabilitation of primary TKA.
2) Whether mobilization exerts better effects than physical modality therapy for primary TKA.
3) The side effects associated with mobilization.

### 2.2 Study method

We will design a single-blind RCT to compare the effects of joint mobilization techniques and physical modality therapy with usual care on TKA patients. A total of 120 patients with early postoperative TKA will be enrolled and investigated in Shanghai Shangti Orthropedic Hospital, Shanghai City, China.

All subjects will receive a questionnaire before the study. The questionnaire will include the following: basic information (eg, age), history of injury, pain (visual analog scale, VAS), knee function [the knee joint Hospital for Special Surgery (HSS) score], and Pittsburgh Sleep Quality Index (PSQI). All participants will sign a consent form before the study.

Subjects who meet the inclusion criteria divided into a 1:1:1 ratio will be randomly selected. After the random distribution, patients with early postoperative TKA will be distributed to a control group (regular training), a physical modality therapy group (physical therapy with regular training), and an intervention group (mobilization with regular training). The study period will last 6 months, including a 4-week intervention and follow-up of 2 to 6 months without intervention. Before intervention, evaluation will be conducted during the 2nd and 4th weeks and during the 3rd and 6th months.

### 2.3 Participants

Inclusion criteria include the following:

1. 50 to 80 years old.
2. With a diagnosis of knee osteoarthritis symptoms and surgical indications.
3. Underwent first unilateral total knee replacement.
4. With the same operation method, normal blood clotting index.
5. Conscious and without cognitive impairment.
6. Not more than 2 weeks after TKA.

Exclusion criteria include the following:

1. With serious cardiovascular disease, neurological disease, osteoporosis and metabolic disease.
2. Suffering from hemophilia, sever diabetes, tumor, or function of blood coagulation disorder.
3. With fracture, dislocation, abnormal structure, and other surgeries.
4. Inability to communicate in Chinese.

### 2.4 Exit criteria and management

Early postoperative TKA patients will be allowed or be required to quit the study if

1. Subject has a demand.
2. Subject develops a serious disease (eg, heart disease).
3. Subject experiences side effects with the treatment.

### 2.5 Interventions

Each group will finish usual training protocol twice a day for 4 weeks, and each section will receive health education before intervention. The subjects will be required to record the time and continuance of usual care protocol.

### 2.6 Interventions group

All participants will undergo joint mobilization technical treatment facilitated by physical therapists. The first type of mobilization is the passive oscillatory movement, which is implemented in different ranges of motion or at the limit of the range. This procedure involves a sustained stretching with or without tiny amplitude oscillations for 30 s or more depending on the patient's feedback and desired effects. Accessory movement, shaft rotation, physiological movement, and combinations of any of these actions may form oscillations or sustained stretches. To eliminate any effect of mobilization, we will adopt joint mobilization in the Maitland level 4 grading method. For example, a tibiofemoral anteroposterior movement or patellofemoral movement may be performed to improve the knee flexion angle. This procedure will involve mobilization from grades I and II, followed by transition to grades III and IV, with every manipulation treatment taking 20 minutes at a time, once a day for 4 weeks.

### 2.7 Physical modality group

The participants will undergo a semiconductor laser device (MDC diode laser system, MDC-1000-IBP) treatment. They will be treated with a laser dose of 6 J/cm^2^ over 8 points around the knee.^[24]^ The selected points are the surgical incision, medial and lateral femoral condyle, patellar up and down, and popliteal space. Laser therapy will be administered at a low power (50 mW, continuous wave, wavelength 880 nm) for 20 minutes at a time, once a day for 4 weeks.

### 2.8 Control group

Participants in the control group will be subjected to regular training, including static quadriceps contraction, straight leg-raising, bridge, ankle pumps, knee joint active movement, and so on. Participants in the intervention group will undergo regular training with joint mobilization, whereas those in the physical modality group will undergo training similar to those in the control group but with physical factors. Regular training takes 20 minutes at a time, 2 times a day for 4 weeks.

### 2.9 Measurement of outcomes

Tools to measure primary indicators include the following:

1. The VAS is used to assess pain intensity. It has a length of 100 mm and a pain scale of 0 to 10, where 0 represents no pain and 10 represents unbearable pain. The pain intensity is determined by the patient.^[25]^
2. The knee joint HSS score, with a 100-point scoring system, is used to gauge knee function. It applies the following criteria: pain, 30 points; function activity, 22 points; range of motion, 18 points; muscle strength, 10 points; flexion deformity, 10 points; and stability, 10 points. A score of ≥85 points is equivalent to best, 70–84 to good, 60–69 to medium, and ≤59 points to poor. The HSS score has become the gold standard to evaluate knee arthroplasty.^[26]^
3. Adverse events associated with joint mobilization technique that will be recorded.

Tools to measure secondary indicators include the following:

1. Manual muscle testing is used to evaluate the knee joint muscle strength.^[27]^ It does not require any equipment when performing strength evaluation of subjects. It applies the following ratings: 0 as zero (O), 1 as trace (T), 2 as poor (P), 3 as fair (F), 4 as good (G), and 5 as normal (N). This procedure evaluates the function and strength of individual muscles based on the effective performance of a movement in relation to the forces of gravity and resistance. This method is simple, easy, and has been widely used in clinics.
2. Joint position matching test is used for knee proprioception.^[28]^ The subject will be asked to move to a reference position (flexion or extension) and maintain in this position for 3 seconds, and then repeat from the starting position to the reference position. The participant will determine the best position and will remain in this posture so that the assessor measures the position and angle. This test will be repeated three times, and the results will be averaged. Greater absolute error corresponds to worse proprioceptive.
3. Berg balance scale has been diffusely used to test the patient's static and dynamic balance abilities. This tool evaluates standing up, sitting down, standing alone, closing one's eyes, raising arms forward, turning, and stepping on one's foot, for 14 times. The ratings of this type of scale are as follows: 0–20 points, balance ability is poor; 21–40, with medium fall risk; and 41–56, with low fall risk.^[29]^
4. In muscle morphology, a musculoskeletal ultrasound is performed with the use of an ultrasonic machine to measure the thickness of the muscle around the knee joint.^[30]^
5. Quality of life will be measured with the SF-36.^[31]^ The SF-36 simplified version includes physical activity and physical function with the role of self-evaluation of health, body pain, overall dynamic, social function, emotional impact on role function and mental health. SF-36 is recognized to be highly reliable in determining quality of life.
6. PSQI^[32]^ scale is used to evaluate the quality of sleep of persons with mental disorders but can also be applied to persons with none. This measure consists of 19 self-evaluation and five other review items. It uses the scores 0–21 with 21 being the highest and implying a poor sleep quality.

### 2.10 Statistical analysis

Statistical analyses will be implemented by SPSS 17.0 and Microsoft Excel 2007 software. Data will be represented as mean ± standard deviation (SD). We will use a 2-way repeated measurement analysis of diversification (group × time) to compute the impact of joint mobilization techniques, physical modality therapy, and the control process, which involve the preliminary and final intervention effects. If subjects fail to make a follow-up, we will use an intention-to-treat analysis. A *t*-test will be performed to compare the changes in measures within groups. Statistical significance will be considered at *P* < .05.

## 3 Discussion

The theory of joint mobilization should be an effective treatment for early TKA. Nevertheless, its effects on early TKA are still controversial. We will perform a single-blind RCT of joint mobilization to patients with early TKA. We believe that the study will provide evidence that joint mobilization can accelerate rehabilitation for primary TKA as compared with physical modality therapy and usual care by decreasing pain and improving range of motion and quality of life.

### 3.1 Strengths and limitations

First, most previous research on joint mobilization typically ranged in persistence from a few hours to 2 weeks.^[33–35]^ The trial duration has a 4-week intervention period and 3 months of follow-up and a total of 6 months of study. Second, previous studies mainly focused on pain, deep vein thrombosis of lower limbs, range of motion, and quality of life.^[36–38]^ Knee proprioception and rectus muscle movement are seldom canvassed for mobilization on early TKA. Third, we set up 3 groups, namely, intervention group, physical modality therapy group, and usual care group, which make the research more rigorous and comprehensive. The limitation of our trial is that it has a lesser number of subjects, with only 120 patients. Moreover, the technique will be performed by different physical therapists. Ideally, to maintain consistency, it should be performed by only one therapist.

In summary, the purpose of this study is to establish the effects of joint mobilization techniques on early TKA patients and to determine whether it generates more favorable outcomes than physical modality therapy or usual care for early TKA. The results of this study will serve as a guide for TKA patients, researchers, and policymaking bodies in their assessment, exclusion, inclusion, and analysis for TKA treatment.
